# Opportunities and challenges in the application of single-cell and spatial transcriptomics in plants

**DOI:** 10.3389/fpls.2023.1185377

**Published:** 2023-08-11

**Authors:** Ce Chen, Yining Ge, Lingli Lu

**Affiliations:** ^1^ Ministry of Education Key Laboratory of Environment Remediation and Ecological Health, College of Environmental and Resource Sciences, Zhejiang University, Hangzhou, China; ^2^ Key Laboratory of Agricultural Resource and Environment of Zhejiang Province, College of Environmental and Resource Sciences, Zhejiang University, Hangzhou, China

**Keywords:** single-cell transcriptomics, spatial transcriptomics, single-nucleus RNA-seq, single-cell RNA-seq, protoplast

## Abstract

Single-cell and spatial transcriptomics have diverted researchers’ attention from the multicellular level to the single-cell level and spatial information. Single-cell transcriptomes provide insights into the transcriptome at the single-cell level, whereas spatial transcriptomes help preserve spatial information. Although these two omics technologies are helpful and mature, further research is needed to ensure their widespread applicability in plant studies. Reviewing recent research on plant single-cell or spatial transcriptomics, we compared the different experimental methods used in various plants. The limitations and challenges are clear for both single-cell and spatial transcriptomic analyses, such as the lack of applicability, spatial information, or high resolution. Subsequently, we put forth further applications, such as cross-species analysis of roots at the single-cell level and the idea that single-cell transcriptome analysis needs to be combined with other omics analyses to achieve superiority over individual omics analyses. Overall, the results of this review suggest that combining single-cell transcriptomics, spatial transcriptomics, and spatial element distribution can provide a promising research direction, particularly for plant research.

## 1 Introduction

A transcriptome is the collection of all transcripts in a cell under specific physiological conditions. Transcriptomics can reveal differences in gene expression under different conditions (Shojaee et al., 2021). From the beginning of transcriptome analysis of various organs to the combination of laser capture microdissection (LCM) and high-throughput sequencing for single-cell transcriptome analysis, the demand for the resolution of transcriptome analysis is increasing. Traditional simple transcriptome analysis cannot meet this demand; however, single-cell transcriptome analysis can. As its name implies, a single-cell transcriptome is the transcriptome of a single cell. Since scientists first reported single-cell transcriptome technology, it has undergone considerable development (Tang et al., 2009). In 2015, two research groups from Harvard developed the Drop-seq and InDrop technologies and applied them to study mouse cells (Klein et al., 2015; Macosko et al., 2015; Klein and Macosko, 2017). In 2016, the 10× Genomics Chromium system, which is a droplet-based technique, was developed. This single-cell sequencing system allows high throughput and can detect rare cell types (See et al., 2018; Svensson et al., 2018; Wang et al., 2021b). Since then, the rapid and low-cost gene expression analyses of many single cells have become a reality. Related single-cell sequencing technologies, such as GemCode single-cell technology (Zheng et al., 2017), MARS-seq2.0 (Keren-Shaul et al., 2019), and Paired-seq (Zhang et al., 2020), are constantly improving in many aspects, such as high cell capture capacity, high gene detection capability, low technical cell-to-cell contamination rate, and low cost, making it possible to use single-cell sequencing technology more conveniently and accurately. Currently, there are three main single-cell sequencing approaches: plate-based, combinatorial indexing-based, and bead-based. Plate-based approaches, e.g., Smart-seq2 and CEL-Seq2, sort a single cell into a well of a multi-well plate; however, these approaches have low throughput and are time consuming. Bead-based methods, such as 10× Chromium, Drop-seq, inDrop, and Seq-Well, use tiny droplets or wells to distribute cells, and are high-throughput and low-cost. Combinatorial indexing-based approaches, such as sciRNA-seq, can reverse transcribe and barcode mRNAs without physically isolating the cells (Ding et al., 2020). Single-cell transcriptomics technology opens a new avenue for molecular studies of tissues and organs from animals and plants, and avoids the lack of information on a particular cell type and heterogeneity from average data (Shulse et al., 2019; Mo and Jiao, 2022).

In plant research, single-cell transcriptomics has developed relatively late, but today it plays an essential and decisive role. Compared to traditional transcriptomics, the sequencing of single plant cells can resolve their heterogeneity. For example, when utilising traditional transcriptomics to profile drought-induced transcriptomic changes, researchers have observed the downregulation of growth-related, energy-consuming processes and the upregulation of stress defence genes at the organ level. However, single-cell transcriptome results suggested that, under mild drought stress, the mesophyll showed significant downregulation of genes, whereas most genes were upregulated in the epidermis. The response of plants to environmental stimuli is complex, and the key to understanding it depends on the study of networks at the single-cell level. The use of single-cell transcriptomics can help pinpoint tissue-specific pathways that respond to stress, and ultimately, these pathways can be specifically engineered in truly important tissues without causing unexpected side effects in other tissues or organs (Tenorio Berrio et al., 2022). There are two sequencing methods: one for sequencing protoplasts and the other for sequencing nuclei. However, the large and uncertain size of plant cells complicates single-cell sequencing. Problems persist even after removing the cell walls to obtain protoplasts, in addition to existing issues with the methods of protoplast extraction (Shulse et al., 2019). These problems can be solved using single nuclei for sequencing instead of whole cells, and the technique has a gene detection sensitivity similar to that of protoplast-based sequencing (van den Brink et al., 2017; Denisenko et al., 2020; Ding et al., 2020; Farmer et al., 2021). Both methods have the disadvantage of losing the spatial information of the cells when isolating them from tissues. Therefore, identifying ways to solve this problem is a widely discussed topic in single-cell transcriptomic research.

Spatial transcriptome technology has undergone considerable development in recent years. This provides a solution that compensates for the shortcomings of single-cell transcription analysis. Spatial transcriptome analysis differs from the method used for single-cell transcriptome analysis and faces specific difficulties. Compared to single-cell and traditional transcriptomics, spatial transcriptomics preserves spatial information while profiling the transcriptome, which provides more precise results for studying regulatory networks in plants. The data obtained from single-cell or traditional transcriptomic analyses are mapped back to the plant through cell sorting instead of using spatial data (Gurazada et al., 2021). During growth, differences in gene expression patterns at different locations in plant tissues lead to different phenotypes. Therefore, spatial transcriptomics provide a new perspective on plant growth and development.

Thus far, there has been a lack of studies to compare the existing methods for plant single-cell transcriptomics, specifically for isolating single cells and nuclei. Studies on spatial transcriptomics in plants are also rare. Here, we introduce and compare in detail the techniques used in related studies in recent years, explain the distinctions among different methods for extracting sequencing materials, and describe the differences between protoplast- and nucleus-based single-cell and spatial transcriptomics. Then, we put forth the challenges and future development directions and provide an idea for combining single-cell and spatial transcriptomics and spatial distribution analysis of elements in plant tissues to promote plant research.

## 2 Development of plant single-cell transcriptomics

Single-cell-related research has been conducted on animals, microorganisms, and plants. Microbiologists have used single-cell-related technologies to explore the differences in RNA accumulation between different parts of microorganisms (de Bekker et al., 2011). Zoologists have used this technology to study tumour cells (Ramsköld et al., 2012) and immune cells (Shalek et al., 2013) or to explore new methods for RNA sequencing (Grindberg et al., 2013). In recent years, plant single-cell transcriptomics has been developed. Researchers have conducted extensive plant studies using single-cell transcriptomic analyses. We reviewed the relevant literature in recent years and tabulated the results (**Table 1**). In 2015, Yan et al. published the first study on whole-genome sequencing of *Zea mays* using single plant cells (Li et al., 2015). Since then, the development of single-cell isolation and sequencing methods has made single-cell transcriptomics a popular topic in botanical research. In 2017, to explore the establishment of monoallelic gene expression in single plant cells, Jiao et al. developed a single-cell transcriptome sequencing protocol and applied it to *Oryza sativa*, which was the first report on the application of single-cell transcriptomics to plants (Han et al., 2017). To date, there have been four single-cell transcriptome studies of plant species: *Arabidopsis thaliana*, *Oryza sativa*, *Solanum lycopersicum*, and *Zea mays*, in the Single Cell Expression Atlas of the European Molecular Biology Laboratory (Conde et al., 2021).

**Table 1:** An overview of plant single-cell transcriptomics related research in recent years.

Plant Species	Positions	Research direction	Publication Year	Reference
*Oryza sativa* L.	middle part of the second leaf	monoallelic gene expression	2017	(Han et al., 2017)
	leaves	a simple system for predicting transcription factor targets	2020	(Xie et al., 2020)
	tips (5 mm) of crown roots	root cell atlas	2021	(Liu et al., 2021b)
	proximal shoots, roots	atlas	2021	(Wang et al., 2021c)
	root tips of rice radicles	atlas (differentiation trajectory)	2021	(Zhang et al., 2021b)
	young inflorescences, leaves	inflorescence development and atlas	2022	(Zong et al., 2022)
*Arabidopsis thaliana*	whole roots	atlas	2019	(Jean-Baptiste et al., 2019)
	primary root tips	atlas (gene expression map)	2019	(Ryu et al., 2019)
	roots	atlas, cell type identification	2019	(Shulse et al., 2019)
	sperm cells	protocol	2019	(Misra et al., 2019)
	root tips	atlas	2019	(Denyer et al., 2019)
	root tips	atlas	2019	(Zhang et al., 2019)
	cotyledons	stomatal lineage cell development	2020	(Liu et al., 2020)
	female gametic cells	polyploid	2020	(Song et al., 2020)
	root tips	properties of the cell cycle	2020	(Torii et al., 2020)
	root tips	atlas, limiting phosphate condition	2020	(Wendrich et al., 2020)
	roots	lateral root development	2021	(Gala et al., 2021)
	root tips	phloem development	2021	(Roszak et al., 2021)
	roots	brassinosteroid signaling	2021	(Graeff et al., 2021)
	roots	optimizing sample size	2021	(Chen et al., 2021a)
	roots	vascular development	2021	(Yang et al., 2021)
	ovule	the female germline differentiation trajectory	2021	(Hou et al., 2021)
	primary roots	atlas	2021	(Lhamo and Luan, 2021)
	roots	regulatory landscape	2021	(Dorrity et al., 2021)
	vascular cells from leaf	atlas	2021	(Kim et al., 2021)
	shoot apexes	atlas	2021	(Zhang et al., 2021c)
	seedlings, developing flowers	nuclei isolation method, atlas	2021	(Sunaga-Franze et al., 2021)
	whole aerial tissue, first true leaves	development	2021	(Lopez-Anido et al., 2021)
	cauline leaves	spatial transcriptome profiles	2022	(Xia et al., 2022)
	leaf explants	*de novo* root regeneration	2022	(Liu et al., 2022c)
	leaves	plant immune system	2022	(Salguero-Linares et al., 2022)
	seedling cotyledons	development of leaf veins	2022	(Liu et al., 2022d)
	roots	root atlas	2022	(Shahan et al., 2022)
	primary roots, above-ground tissues	transcriptome profiles (atlas)	2022	(Apelt et al., 2022)
	the third leaf	atlas	2022	(Tenorio Berrio et al., 2022)
*Zea mays* L.	root hairs, primary roots without root hairs	development, gene identification	2017	(Hey et al., 2017)
	anthers	development	2019	(Nelms and Walbot, 2019)
	shoot apical meristem	developmental genetic organization	2020	(Satterlee et al., 2020)
	ears	developmental atlas	2021	(Xu et al., 2021a)
	anthers	cytoplasmic male sterility	2021	(Zhang et al., 2021a)
	seedlings, tassel or ear primordia, root tips, crown roots, axillary buds, whole roots	cis-regulatory atlas	2021	(Marand et al., 2021)
	anthers, pollens	haploid induction key gene molecular mechanism	2022	(Jiang et al., 2022)
*Gossypium hirsutum*	fiber	photoinduced fiber color formation, molecular regulatory	2021	(Tang et al., 2021)
	root tips	method, atlas, salt stress	2022	(Liu et al., 2022b)
*C. sinensis* var. *sinensis*	leaves	atlas	2022	(Wang et al., 2022)
*Populus alba*	stems	vascular development	2021	(Chen et al., 2021b)
*Triticum aestivum* L.	coleoptile	atlas, the turgor alteration of guard cells	2021	(Wang et al., 2021a)
*Arachis hypogaea* L.	leaves	method, atlas, developmental trajectory and interaction network	2021	(Liu et al., 2021a)

Published studies on plant single-cell transcriptomics are listed in this table. The plant species, the position of plant tissues used in these studies, research directions, and publication years are listed in the table.

From the published studies listed in **Table 1**, it is clear that most experiments were based on *A. thaliana*, and subsequently on *O. sativa* and *Z. mays*. Scientists typically use whole plant roots and root tips as experimental materials. A few scientists have also used other plant parts, such as leaves, stems, or germ cells, based on their research direction and purpose. **Table 1** shows that most scientists have mainly constructed a single-cell atlas to explore the cell types of particular parts of plants, specifically roots, and examined developmental or differentiation trajectories combined with trajectory analysis, as transcriptomics can deliver information about gene expression.

Single-cell transcriptome technology has been used in numerous plant studies. In 2019, a group studied cell types from the primary root tissues of *A. thaliana* using a high-throughput Drop-seq approach. Their findings confirmed that RNA sequencing helps describe the developmental processes of plants (Shulse et al., 2019). It is worth mentioning that they also revealed that external stimuli, such as sucrose, can influence the developmental process, causing changes in sucrose response-related cells. In a similar case, in 2022, a group studied another external stimulus, salt stress, using cotton as their object and observed the influence of external stimuli on the profile and dynamic changes in gene expression (Liu et al., 2022b). For further research on the exploration of cellular heterogeneity, it is essential to compare gene expression profiles before and after external environmental stimuli by studying single-cell transcriptome sequencing results and constructing an atlas. In addition to investigating the environmental influences on plant cell gene expression, single-cell transcriptomics is a hot topic in the study of plant development and differentiation. One group has built models of cell differentiation within leaf tissues, studying stomatal lineage, and thereby determined a series of cellular programs related to tissue flexibility (Lopez-Anido et al., 2021).

With the help of single-cell transcriptomics, scientists have explored gene expression and signal transduction pathways while studying cell developmental trajectories. However, no research has been conducted on this topic. For example, researchers have observed how cytokinin signalling creates a link between the vascular perception of limited phosphate availability and epidermal responses (Wendrich et al., 2020). They drew an intersection between a single-cell atlas and target genes. They observed the distribution of transcription factor complexes in specific tissues, revealing the significance of the cytokinin signalling pathway. Comparing single-cell transcriptomic atlas and target genes in a critical signalling pathway, such as brassinosteroid signalling (Graeff et al., 2021), helps to understand the functions or effects of particular gene products or signal complexes in plants and their delivery or distribution in different tissues. Many scientists have used single-cell transcriptome techniques to create an atlas of plant species, which still have some genes that need to be explored, and have compared them with those of *A. thaliana* to study the functions of a few genes. This comparison reduces the difficulties and increases the hope of constructing a cell atlas for different plant species. However, studies involving target genes are limited, and even fewer studies have examined gene functions. In future studies, combining the analysis of an atlas with the target genes will be an analytical method that will attract increasing attention.

In addition to single-cell transcriptomics, there are many other omics and single-cell omics studies, and a few scientists have combined single-cell transcriptomic analysis with other omics analyses. For example, plant cells can be isolated for single-cell transcriptomics, which results in the loss of spatial information. Therefore, other omics techniques, such as spatial transcriptomics, are required to supplement missing data. In 2022, scientists applied scStereo-seq technology to plant research and constructed a single-cell spatial atlas (Xia et al., 2022). Owing to the combination of two omics technologies, they avoided a few disadvantages of a single omics technology and observed that the expression levels of related genes showed a gradient change trend in space. Another group combined single-cell transcriptomics technology with multi omics technology to study the molecular basis of developing *Z. mays* ears (Xu et al., 2021a). In addition to combining single-cell transcriptomics with spatial transcriptomics, some scientists have applied single-nucleus RNA sequencing and ATAC sequencing to plant roots, compared transcriptomic and epigenomic data at the single-cell level, and revealed how chromatin accessibility influences gene expression (Farmer et al., 2021). Using different omics technologies in combination with single-cell transcriptomics helps researchers solve problems associated with using only one technology, such as gene redundancy and spatial information.

As mentioned above, plant cells have cell walls, which make it challenging to obtain a single cell. Isolating single cells is affected by many factors, such as the parts or tissues of the plant used and the environmental conditions or genotypes of the plants (Shaw et al., 2021). Therefore, few researchers have been concerned with the methods used to perform single-cell transcriptomics. They introduced a commonly used method for isolating plant nuclei and demonstrated its universal applicability (Sunaga-Franze et al., 2021). A few researchers have introduced protoplast isolation methods for cotton (Liu et al., 2022b). New methods, not only for single-cell extraction but also for sequencing, have significance in plant research, reducing costs and obtaining enough single cells.

## 3 Standard methods for single-cell transcriptomics in plants

Sequencing of plant single-cell transcriptomes can be divided into sequencing of protoplasts and sequencing of nuclei. More accurately, the latter should be referred to as the single-nucleus transcriptome. Comparing the sequencing processes of the two, overall, the sequencing materials are all obtained from plant tissues, and then purified and separated for sequencing. Specifically, to protect the activity and integrity of protoplasts, the methods used are gentler and require higher freshness of plant samples during the process of obtaining protoplasts, whereas frozen or dried plant samples can be used to obtain nuclei. The following is a detailed introduction to both methods.

**Figure 1:**
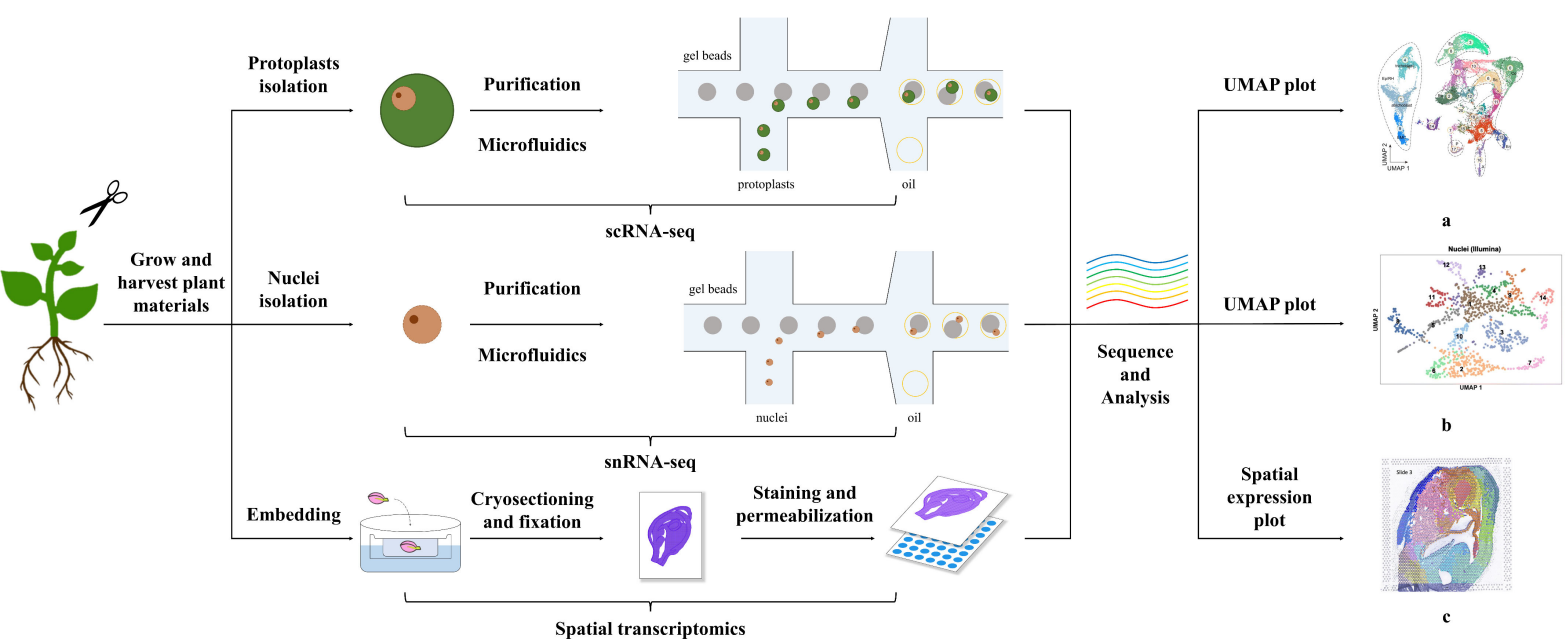
Methods of Single-Cell Transcriptomics and Spatial Transcriptomics in Plants. The figure shows the general process for performing single-cell and spatial transcriptomics. Single-cell transcriptome technologies are divided mainly into protoplast-based and nucleus-based methods. Both approaches include collecting plant materials, separating protoplasts/nuclei through microfluidics after purification steps, i.e., filtration, centrifugation, and washing, confirming the viability and concentration of obtained protoplasts or cell nuclei, and sequencing and analysis. Spatial transcriptome methods differ from single-cell transcriptome methods and include embedding, cryosectioning, fixation, staining, and permeabilization steps. The plots a, b, c shown in the figure are visual diagrams of analysis results of protoplast-based sequencing, nucleus-based sequencing, and spatial transcriptomics sequencing respectively from published studies (Long et al., 2021; Zhang et al., 2021b; Liu et al., 2022a). The plots of single-cell transcriptomics, such as uniform manifold approximation and projection (UMAP) plots, present the cell classification results of sequencing, and those of spatial transcriptomics demonstrate the combination of transcriptome and spatial information.

**Figure 2:**
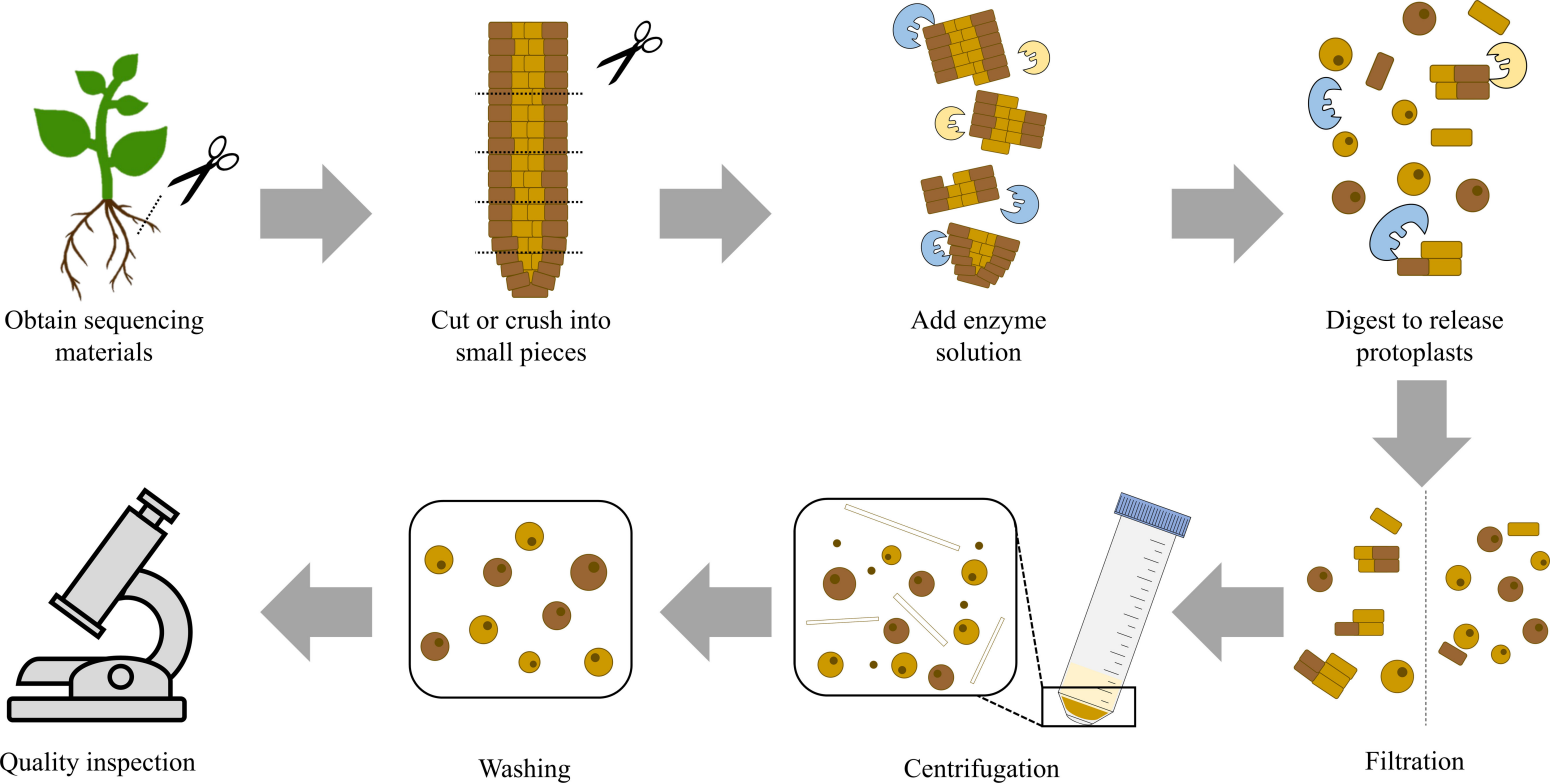
Flow Chart for Protoplast Isolation. The figure shows the general process for protoplast isolation. First, obtain sequencing materials and cut or crush them into small pieces. Then, add the prepared enzyme solution. The amount and proportion of enzymes need to be appropriate. The reaction is carried out under appropriate conditions and enzymes digest plant cell walls to release protoplasts. This step is followed by further steps that involve filtration, centrifugation, and washing. Finally, the quality and quantity of the obtained protoplast solution are tested. If both meet the expectations, the preparation of the protoplast solution is completed.

Based on recent protoplast-based single-cell transcriptomics studies (**Table 1**), it is clear that these procedures are similar (**Figures 1**, **2**). First, the plants were grown based on the research goals. For example, to explore the single-cell transcriptome of rice roots under Cd stress, a small amount of rice was planted in both standard and Cd-stress environments. After a period of growth, the plant material is harvested from the required parts, including the root tips, whole roots, leaves, or other parts of the plant, and cut or crushed into small pieces to expand the area of the plant material exposed to the enzyme solution and shorten the time required for enzyme treatment. Plant materials are then mixed with the enzyme solution, called the digesting enzyme solution, and incubated under appropriate conditions to release protoplasts or plant cell nuclei. Plant materials must be mixed with an enzyme solution as soon as possible after being cut or crushed to maintain cell activity and improve the quality of protoplasts. This step is followed by further steps that involve filtration, centrifugation, and washing to obtain sufficient material for sequencing, relatively fewer undigested plant cells, and fewer broken cell organelles. Digestion helps remove the cell wall to isolate protoplasts, filtration helps remove substances that are not protoplasts or are larger in size, centrifugation helps concentrate protoplasts, and washing helps remove impurities and residual reagents. The quality and concentration of sequencing materials must be confirmed, as high quality and quantity are the basis for further sequencing to obtain sufficiently precise transcriptomic data (Liu et al., 2022b). A single-cell RNA sequencing (scRNA-seq) library is prepared and sequenced. Finally, the data are analysed to obtain the experimental results. This is the entire process of using single-cell transcriptomics; however, when it comes to each specific experiment, there still exist differences, particularly in the methods used to obtain ideal protoplast materials. A critical step in protoplast-based single-cell transcriptomics in plants is obtaining high-quality sequencing objects. Culture conditions, genotype, plant age, and other factors affect extraction methods to varying degrees, implying that when preparing protoplasts, the procedures must be adjusted based on the experimental conditions. In **Table 2**, the methods of partial studies are compared, that is, from studies listed in **Table 1** or studies the authors referred to if they did not write the procedure for obtaining protoplasts.

**Table 2:** Comparisons between methods used for protoplast-based single-cell transcriptome analysis in partially published studies.

Species	Position	Composition of enzyme solution (generally used)	Composition of enzyme solution (optionally used)	Optional incubation condition	Optional centrifugation condition	Optional washing solution	Optional sequencing protocol	Reference
*Oryza sativa* L.	roots, proximal shoots	1. BSA 2. CaCl_2_ 3. Cellulase 4. Macerozyme 5. Mannitol 6. MES	1. Hemicellulase 2. KCl 3. NaCl 4. Mercapto-ethanol 5. Pectolyase	1. 10–30 min at vacuum pump at room temperature and then 2–2.5 h at room temperature (about 28 °C) with shaking at 50–70 rpm	1. 130 g for 5 min 2. 100 g for 3 min	1. 8% mannitol	1. 10× Genomics 2. BD Rhapsody system 3. Cel-Seq2 4. SMART-seq	(Han et al., 2017; Xie et al., 2020; Liu et al., 2021b; Wang et al., 2021c; Zhang et al., 2021b; Zong et al., 2022)
	leaves, inflorescences			1. 2-3 h at room temperature	1. 200 g for 3 min	1. 8% mannitol 2. PBS-BSA		
	leaves			1. 3 h at 28 °C with shaking at 70 rpm in the dark	1. 300 g	1. W5 solution		
*Arabidopsis thaliana*	roots	1. BSA 2. CaCl_2_ 3. Cellulase 4. KCl 5. Macerozyme 6. Mannitol 7. MES	1. Actinomycin D 2. Cordycepin 3. DTT 4. HCl 5. Mercapto-ethanol 6. MgC_l2_ 7. Pectolyase	1. 1 h at room temperature with shaking at 75–85 rpm 2. 1 h at 75 rpm (1 g whole roots and 10 mL enzyme solution) 3. 2 h at 20 °C at 200 rpm 4. 1 h at room temperature at 75 rpm (1,500 seedling roots and 10 mL enzyme solution)	1. 500 g for 5−10 min 2. 200 g for 6 min	1. 8% mannitol 2. Protoplast solution without enzymes 3. W5 solution		(Yoo et al., 2007; Bargmann and Birnbaum, 2010; Denyer et al., 2019; Jean-Baptiste et al., 2019; Ryu et al., 2019; Wendrich et al., 2020; Gala et al., 2021; Hou et al., 2021; Kim et al., 2021; Lopez-Anido et al., 2021; Yang et al., 2021; Zhang et al., 2021c; Apelt et al., 2022; Liu et al., 2022c; Liu et al., 2022d; Shahan et al., 2022; Tenorio Berrio et al., 2022)
	leaves			1. 30 min at room temperature 2. 2 h with gentle shaking (whole aerial tissue or first true leaf and 15 mL enzyme solution) 3. 30 min in the dark with vacuum treatment and then 3 h at room temperature (10–20 leaves in 5–10 ml enzyme solution) 4. 2 h at 30 rpm	1. 500 g for 5–10 min 2. 100 g for 1–7 min	1. W5 solution 2. protoplast solution without enzymes		
	shoot apexes, leaves			1. 2 h at room temperature	1. 500 g for 5–10 min	1. 8% mannitol		
	cotyledons			1. 10 min for vacuum infiltration and then 4 h for incubation				
	ovule			1. 3 h at 100 rpm		1. protoplast solution without enzymes		
*Zea mays* L.	shoot apical meristem	1. BSA 2. CaCl_2_ 3. Cellulase (Cellulase Onozuka R-10, Cellulase Onozuka RS) 4. Hemicellulose 5. HCl 6. KCl 7. Macerozyme 8. Mannitol 9. MES 10. Pectolyase	1. MOPS 2. β-mercaptoethanol	1. 2 h at 29 °C with gentle shaking	1. 250 g for 3 min	1. Washing buffer (0.65 M mannitol, 10 mM MOPS pH7.5 and 10 mM L-Arginine, at pH 7.5)		(Ortiz-Ramirez et al., 2018; Satterlee et al., 2020; Xu et al., 2021a)
	ears, roots			1. 45 minutes at room temperature with gentle shaking	1. 500 g for 3 min	1. Washing solution (1.82 g mannitol, 0.097 g MES, 1 M KCl, 1M CaCl_2_, 0.025 g BSA, adjust pH solution to 5.7 with 1M TRIS)		
*Gossypium hirsutum*	roots	1. BSA 2. CaC_l2_ 3. Cellulase 4. KCl 5. Mannitol 6. MES 7. Pectolyase		1. 1 h under dark with vacuum treatment and then 6 h at 25 °C under dark at 80 rpm		1. WB buffer		(Liu et al., 2022b)
*C. sinensis* var. *sinensis*	leaves	1. BSA 2. CaCl_2_ 3. Cellulase 4. KCl 5. Macerozyme 6. Mannitol 7. MES	1. Snailase 2. β-mercaptoethanol	1. 5 min with vacuum treatment and then 4 h at 25 °C under dark with gentle shaking	1. 100 g for 2 min	1. W5 solution		(Xu et al., 2021b; Wang et al., 2022)
	fresh samples			1. 30 min under the pressure of −0.1 MPa and then at 25 °C with gentle shaking at 45 rpm under dark (1 g fresh weight samples and 10 mL enzyme solution)	1. 200 g for 3 min			
*Populus alba*	stems	1. BSA 2. CaCl_2_ 3. Cellulase 4. KCl 5. Macerozyme 6. Mannitol	1. MES	1. 2.5 h in the dark at room temperature with shaking at 50–55rpm 2. 20 min in the dark at room temperature and then gently shake by hand for 30 s (four 10 cm stem segments and 40 mL enzyme solution)	1. 100 g for 4 min	1. W5 solution		(Lin et al., 2014; Chen et al., 2021b)
*Triticum aestivum* L.	guard cells	1. Actinomycin D 2. CaCl_2_ 3. Cellulase 4. Cordycepin 5. KH_2_PO_4_ 6. Macerozyme 7. MES 8. MgCl_2_ 9. RNase inhibitor		1. 1.5 h at 25 °C				(Wang et al., 2021a)
*Arachis hypogaea* L.	leaves	1. BSA 2. Cellulase 3. Macerozyme 4. Mannitol (without Ca^2+^ and Mg^2+^) 5. MES 6. Pectinase		1. 2 h at 25 °C with shaking at 40 rpm		1. 8% mannitol		(Liu et al., 2021a)

The compositions of enzyme solutions, incubation conditions, centrifugation conditions, the types of washing solutions, and sequencing protocols used in partially published studies are listed in this table.

1. If the enzyme solution composition is used in more than half of the references, we consider it as generally-used composition.

2. The solution needs to be heated in a warm bath at approximately 55 °C after adding part of materials (enzymes, etc.) and then, after cooling to room temperature (approximately 25 °C), the solution is added in the rest materials.

3. If the article has mentioned the ratio of plant materials and enzyme solution, we will mark it in brackets.

First, the enzyme solution used during protoplasting differs among the different methods, but the types of reagents and enzymes used are similar in some methods. Enzymes are used to digest cell walls, whereas other reagents primarily serve to maintain the state of protoplasts or the efficiency of enzymes. Plant cell walls are mainly composed of cellulose, hemicellulose, and pectin, and the corresponding enzymes, including cellulase, pectolyase, and hemicellulose, are used to digest the cell walls (Somssich et al., 2016). However, most studies have used only cellulase and macerozymes, as macerozymes contain pectolyase and hemicellulase activities. A few scientists have added all four types of enzymes, that is cellulase, macerozyme, hemicellulose, and pectolyase, to the solution to obtain protoplasts from rice (Liu et al., 2021b). A few scientists have also added another enzyme, snailase, to reduce digestion time (Wang et al., 2022). Adding more enzymes is conducive to shortening the enzymatic hydrolysis time but can cause enzyme waste. The composition of cell walls differs among different plant species and organs. Therefore, determining the appropriate enzyme content and ratio is conducive for efficient enzymatic digestion. Bovine serum albumin (BSA), CaCl_2_, mannitol, and 2-(N-morpholino)ethanesulfonic acid (MES) are generally used to protect protoplasts. An enzyme solution with added BSA has the benefit of protecting the protoplasts, and a few researchers have used dithiothreitol (DTT) to perform the same function as BSA (Kim et al., 2021). Although BSA seems necessary to stabilise the enzyme solution, few researchers have used it (Xie et al., 2020). Salts such as CaCl_2_ are used to maintain the osmotic pressure of protoplasts *in vivo* and *in vitro* and prevent protoplasts from absorbing water and breaking. There do exist differences—for example, when studying *A. thaliana* and *Z. may*s, researchers are more likely to add KCl into the enzyme solution than when studying *O. sativa* as shown in **Table 2**. There are still differences in details within the same species, even when using the same parts of the materials in different studies. For example, when preparing rice protoplasts, a few studies added KCl to the digestion solution, whereas several other studies did not; however, mercaptoethanol was added (Han et al., 2017; Liu et al., 2021b; Wang et al., 2021c). NaCl has been added to enzyme solutions in a few studies (Xie et al., 2020). In addition, **Table 2** shows differences among the types of mercaptoethanol used in solution: a few used 2-mercaptoethanol, others used 4-mercaptoethanol acid, and a few others used β-mercaptoethanol.

After determining the composition of the enzyme solution, reagents and enzymes were mixed. Usually, scientists dissolve cellulase, macerozyme, mannitol, MES, and certain enzyme solution reagents in water and heat the mix to activate enzymes in a warm bath at approximately 55 °C for nearly 10 min. Upon heating, the turbid enzyme solution becomes clear. After heating, it is cooled to room temperature, i.e., approximately 25 °C, and the rest of the materials, including BSA, CaCl_2_, and mercaptoethanol, are added before adding plant materials. Unlike the general methods of mixing plant materials and the entire digestion solution, a few techniques involve mixing plant materials and the enzyme solution and incubating the mixture in the dark for the reaction. The remaining chemicals, including NaCl, CaCl_2_, KCl, and MES, are added for further enzymatic reactions to release protoplasts (Yoo et al., 2007; Xie et al., 2020; Gala et al., 2021).

After mixing the enzyme solution with plant materials, it is essential to provide sufficient time and appropriate reaction conditions to ensure the release of protoplasts. Most studies listed in **Table 2** did not specify the proportions of plant materials and enzyme solutions. **Table 2** lists the incubation conditions. A few groups used a vacuum pump to isolate protoplasts in experiments to reduce the digestion reaction time, whereas others did not. Therefore, the reaction times of others were longer than reaction times of those that used the vacuum pump. This is because negative pressure promotes the infiltration of the enzyme solution into plant materials, such that the contact surface between the enzyme and plant cells increases, thereby reducing the reaction time. In addition, it is helpful to incubate a mixture of enzyme solution and plant materials in the dark or to shake this mixture using a machine or manually to reduce the reaction time. When shaking the enzyme solution, it is necessary to shake it gently at a speed of 40–80 rpm to avoid cell fragmentation caused by violent shaking. However, a few groups used 200 rpm shaking speed (Denyer et al., 2019). The reaction times used in different studies to release protoplasts are similar, but there are still differences in the details. For example, when digesting the leaves of *A. thaliana*, the digestion time used by a few groups was 30 min, whereas others chose to digest for 2 h, probably because the leaves they used were different; that is, leaves from the leaf base regions or the third leaf (Liu et al., 2022c; Tenorio Berrio et al., 2022). Although different digestion times are listed in **Table 2**, it is only for reference—digestion times can differ based on differences in the digestion conditions, species, and parts of plants used, and it is necessary to check whether most of the plant cell walls are digested at least every half an hour. If measures are taken to shorten the time, the enzyme content in the enzyme solution will be higher, and if the plant tissues used are young or have a short growth time, the digestion time will be shorter.

After protoplast release, cells are filtered, centrifuged, and washed. Filtration aims to remove undigested plant cells and obtain protoplasts; therefore, the filter must be of the proper size. The size of plant protoplasts is small, e.g., *A. thaliana* mesophyll protoplast diameter ranges from 30 to 50 μm, and therefore, most groups used 30–80 μm nylon mesh or cell strainer to filter protoplasts (Yoo et al., 2007). A few groups filtered the protoplasts only once, whereas other groups filtered them twice or more times, and other groups filtered the protoplasts through two different sizes of filters to obtain more protoplasts and less undigested plant cells (Wang et al., 2021c; Zhang et al., 2021b; Liu et al., 2022c). Centrifugation helps concentrate protoplasts, and the centrifugation conditions differ among different plant species. **Table 2** lists the centrifugation conditions used in various studies. Centrifugation speed and time depend on the characteristics of the plant materials, such as their fragility, type, and the number of cells produced during digestion (Bargmann and Birnbaum, 2010). High centrifugation speed can increase both the recovery rate of protoplasts and the risk of cell disruption (Yoo et al., 2007). As shown in **Table 2**, when centrifuging rice protoplasts, the centrifugation speed (e.g., 100 g, 130 g, or 200 g) is slower and the centrifugation time (e.g., 3 min or 5 min) is shorter than that used for centrifuging *A. thaliana* (e.g., 500 g for 5–10 min). The differences in centrifugation speeds may result from the different characteristics of the protoplasts of these two plants or from differences in the isolation steps used in published studies. During centrifugation, a few groups maintained the solution at constant low-temperature conditions, such as 4 °C or −4 °C, to protect protoplasts from damage (Denyer et al., 2019; Satterlee et al., 2020; Xu et al., 2021a). It is also possible to collect small protoplasts by centrifugation at low speeds and then increase the speed of collecting larger protoplasts, avoiding the breakage of small protoplasts and the loss of large protoplasts caused by insufficient centrifugation speeds. Washing is also necessary because it can help remove specific ions or tissue residues that affect the downstream steps, such as undigested plant tissues and Mg^2+^ ions (Liu et al., 2022d). During the preparation of the washing solution, the entry of impurities is avoided, and an appropriate osmotic pressure amenable to protoplasts is maintained in the washing solution. Scientists have typically used 8% mannitol or a protoplast solution without enzymes for washing; however, a few scientists have also used W5 solution, WB buffer, or other washing buffers (**Table 2**). It is worth mentioning that the components of W5 solutions prepared by different groups showed little difference (Chen et al., 2021b; Xu et al., 2021b). After washing, the protoplasts were centrifuged again to separate the washing solution from the protoplasts. The centrifugation speed and time can be the same as those used in the centrifugation step, or at a slower rate, shorter time, or both. The protoplasts are resuspended in mannitol, washing solution, or other solutions based on the sequencing protocol, which helps adjust the concentration of protoplasts and avoid influencing reverse transcription reactions (Chen et al., 2021b). The viability and concentration of the protoplasts are then confirmed for better sequencing results. Trypan blue, 4′,6-diamidino-2-phenylindole, fluorescein diacetate, or acridine orange-propidium iodide are used to select protoplasts, and a haemocytometer is used to measure the protoplast concentration. The viability and concentration of protoplasts need to be adjusted to reach the standard of sequencing methods, and the concentration of protoplasts can be adjusted using a resuspension solution. Next, an scRNA-seq library is prepared and sequenced according to different protocols.

The process of isolating plant nuclei is similar to that used to separate plant protoplasts. The plant material used to extract nuclei can be either fresh or frozen. Plant tissues are cut or crushed into small pieces and mixed with a nuclei extraction solution. The solutions used for nuclei extraction in the relevant studies are listed in **Table 3**; there are similarities, with a few differences. Compared to isolating protoplasts, plant nuclei isolation requires the lysis of plant cell membranes and protection of nuclear membranes; therefore, the solutions are relatively more destructive. The incubation is usually performed on ice. After incubation, filtration, centrifugation, and resuspension are conducted. The speed of centrifugation for isolating plant nuclei is usually faster than 1,000 g; for example, a few groups centrifuge at 2,000 g, whereas others centrifuge at 25,000 g (Giuliano et al., 1988; Dorrity et al., 2021). Plant nuclei are washed, recovered, and resuspended in a buffer solution. Some groups have conducted Percoll gradient centrifugation for impurity removal and nuclear recovery (Dorrity et al., 2021). The remaining steps are performed according to the nucleus-based sequencing protocols of the sequencing platforms.

**Table 3:** Nuclei extraction solutions.

Species	Composition of solution	Reference
*Arabidopsis thaliana*	1. Sucrose 2. MgCl_2_ 3. Tris·HCl 4. Protease Inhibitor Tablet	(Dorrity et al., 2021)
	1. Ficoll 400 2. Dextran T40 3. Sucrose 4. MgCl_2_ 5. DTT 6. Triton X-100 7. cOmplete Protease Inhibitor Cocktail 8. RiboLock 9. Tris HCl	(Sunaga-Franze et al., 2021)
	1. Ficoll 400 2. Dextran T40 3. Sucrose 4. Tris 5. MgCl_2_ 6. Triton X-100 7. β-mercaptoethanol	(Moreno-Romero et al., 2017)
*Zea mays* L.	1. Tris 2. EDTA 3. Spermine 4. KCl 5. NaCl 6. 2-ME 7. Trixton X-100	(Marand et al., 2021)
*Solanum lycopersicum*	1. Sucrose 2. MgCl_2_ 3. Tris·HCl 4. Phenylmethylsulphonyl fluoride 5. Benzamidine 6. 2-mercaptoethanol	(Giuliano et al., 1988)

Different kinds of nuclei extraction solutions used for isolating nuclei in nucleus-based single-cell transcriptomics are listed in this table.

## 4 Development of plant spatial transcriptomics

Spatial transcriptomic technologies originated in mammalian systems and are widely used in mammals. A few published studies have applied omics technology to plant research. However, more strategies are available for mammalian spatial transcriptomics than for plants, mainly because of the differences between animal and plant cell structures such as cell walls. Currently, research on plant spatial transcriptomics is divided primarily into two categories: finding ways to use and optimise spatial transcriptomic methods, and using spatial technology to solve problems in plant research.

In 2017, Stefania and his group provided a method for generating and studying high-resolution and spatially resolved functional profiles in plants (Giacomello et al., 2017; Giacomello and Lundeberg, 2018). At the method level, research has not been limited to methodology in plants or the application of methods used in mammalian systems to plants, but has also focused on finding ways to improve spatial transcriptome technology in plant and mammalian systems. Spatial transcriptome analysis requires high spatial resolution and high spatial transcriptome data, such as maps in daily life, and provides an extensive range of accurate information on buildings located in an area. A few analytical techniques, such as single-cell transcriptomics, produce high-quality data, implying that they provide relatively high spatial resolution but lose spatial information. In addition, sorting or isolation technologies, such as fluorescence-activated cell sorting (FACS), isolation of nuclei tagged in specific cell types (INTACT), and LCM, provide spatial resolution or cell-specific expression data. However, these methods have certain limitations. Plant materials must be transgenic using FACS and INTACT; however, this is a challenge for a few plant species. In addition, many factors such as cell type, yield, and purity affect the quality of the LCM and FACS methods. Technologies such as FACS require the digestion of plant cells to isolate protoplasts, which causes loss of spatial information (Giacomello et al., 2017; Gurazada et al., 2021). Fluorescent *in situ* hybridisation (FISH) is an optimal option for obtaining spatial information from plant cells; however, it can only detect one gene at a time, limiting its speed (Tirichine et al., 2009; Gurazada et al., 2021). Therefore, spatial transcriptomics technology needs to be improved. Currently, there are two main types of spatial transcriptome technology: next-generation sequencing (NGS)- and imaging-based approaches. NGS-based methods involve high-throughput sequencing based on scRNA-seq technology and a spatial barcode, whereas imaging-based methods include *in situ* sequencing (ISS) and *in situ* hybridisation (ISH). Recent research on spatial transcriptomic technologies, including ISH-based MERFISH and ISS-based STARmap, has promoted genetic and genomic studies (Moffitt et al., 2018; Wang et al., 2018; Liu et al., 2022a). Spatial indexing approaches, including 10× Visium, use NGS-based methods to quantify the gene expression profiles formed by the local hybridisation of barcodes and RNA molecules, and the spatial information is stored by barcodes. These approaches can detect the entire transcriptome in an unbiased manner without knowing the target gene in advance but must ensure the integrity of mRNA in the tissues. ISS- and ISH-based methods can intuitively analyse data at organisational spatial locations, but mRNA abundance *in situ* is low and often degraded (Elhanani et al., 2023). Although spatial transcriptomic methods face several challenges, including resolution and sensitivity, researchers are constantly attempting to overcome these difficulties (Rao et al., 2021).

Spatial transcriptomics technologies provide new insights into plant growth, development, and molecular biology. A few researchers have mapped and visualised the related gene expression in C4 and crassulacean acid metabolism in *Portulaca* at the spatial level using the 10× Genomics Visium spatial transcriptomics platform (Moreno-Villena et al., 2022). One group combined spatial and temporal transcriptome information to explore the early development of tomatoes (Zhang et al., 2016). In 2022, a Chinese group studied *Phalaenopsis* Big Chili using 10× Visium technology (Liu et al., 2022a). A few studies have analysed transcriptome data at the spatial level; however, in these studies, researchers collected plant materials in spatial order and then sequenced them separately, which preserved the spatial information and provided help in the analysis, but the information lacked high resolution (Guo et al., 2021).

Spatial transcriptome technology is helpful, but there are still few studies using this technology in plant research owing to many reasons and challenges that will be discussed later.

## 5 Methods used in plant spatial transcriptomics

In plant research, technology for preserving spatial information is not widely used. This is partly because appropriate methods have not been used in plants, and the structures of plant cells differ from those of animal cells, such as the presence of cell walls and vacuoles. In a few studies, the processes involved collecting plant materials in spatial order, separating leaves from outer to inner, sorting root and aerial tissues, and sequencing them separately (Guo et al., 2021; Apelt et al., 2022). Such methods are usually not highly resolved because they involve the sequencing of many mixed cells, providing a considerably limited variety of spatial locations. One such process involves dissecting different tissues for transcriptome analysis, such that the spatial information for the entire section is maintained, but the spatial information is distinguished according to the initially selected regions (Stahl et al., 2016).

A few scientists achieved high-resolution gene expression data, even near single-cell resolution, using high-throughput methods. In general, these methods include collecting targeted sequencing materials, embedding, cryosectioning, fixation, staining, imaging, permeabilisation, and sequencing, according to the manufacturer’s instructions (**Figure 1**) (Giacomello et al., 2017; Giacomello and Lundeberg, 2018; Kivivirta et al., 2019; Liu et al., 2022a; Moreno-Villena et al., 2022). Embedding is usually the first step in spatial transcriptomic experiments after harvesting the targeted plant materials. The samples are embedded in cold optimal cutting temperature (OCT) compound to support the plant tissues during cryosectioning. Plant tissues have structures such as hard cell walls and large vacuoles with high water content, which increase the hardness of plant tissues after conventional freezing, making it difficult to obtain high-quality slices. Then, the plant samples are sliced at the proper temperature (−15 to −20 °C). The thickness of the sections must also be appropriate to avoid breakage and overlapping of the different tissue sections during slicing. In addition, the organisational structure of plant samples is complex, the degree of lignification varies, and the relative RNA content of the tissue is relatively low; therefore, the thickness of plant slices generally depends on the type of sample (Giacomello and Lundeberg, 2018; Liu et al., 2022a). Subsequently, the plant sections are fixed on the array at approximately 37 °C. The solutions used in the fixation step differed among different studies; a few studies used neutral formaldehyde, whereas others used methanol. The dyes used in the staining step also differ among various studies, such as toluidine blue or haematoxylin-eosin staining, and the staining time is usually 1 min, followed by imaging of the stained sections. Permeabilisation is a critical step in spatial transcriptomics following imaging. In this step, it is necessary to determine the optimum permeabilisation conditions for plant samples to release sufficient mRNA (Moreno-Villena et al., 2022). A few experiments have also included a pre-permeabilisation step before permeabilisation for partially denaturing proteins to improve the efficiency of permeabilisation (Giacomello and Lundeberg, 2018). After permeabilisation, the samples were sequenced according to the manufacturer’s protocol, including reverse transcription, tissue removal, and probe release. Finally, the water used in these experiments must be nuclease-free in specific steps to avoid degradation of the cDNA-mRNA hybrids.

Although differences exist between different methods and there are only a few relevant studies in plant research, we can learn from the methods used in mammalian systems or follow the manufacturer’s instructions to optimise these methods in plant systems (Giacomello and Lundeberg, 2018).

## 6 Comparisons among protoplast-based and nucleus-based single-cell transcriptomics and spatial transcriptomics

Although protoplasts and plant nuclei can be used for single-cell sequencing, there are differences, and both have advantages and disadvantages. Comparing these two sequencing methods and the scope of their application is beneficial for attaining a deeper understanding of single-cell transcriptomics in plant systems and for exploring future research directions.

As shown in **Tables 2**, **3**, enzyme solutions, centrifugation speeds, and other parameters differ between the two methods. For example, because of differences in sequencing materials, these two methods use different solutions to digest or isolate plant cells. When separating protoplasts, researchers usually use enzymes to digest the cell walls of plant cells and add various reagents to protect the cell membrane and maintain protoplast shape. In contrast, when isolating nuclei, researchers usually do not use enzymes, but instead use other reagents, such as Triton X-100, to separate plant nuclei, and the cell membranes need to be destroyed (Sikorskaite et al., 2013).

When separating protoplasts, artefacts affecting the transcriptome are introduced by the addition of enzymes. Scientists have confirmed that protoplasts affect transcriptomics (Birnbaum et al., 2003; Denisenko et al., 2020; Conde et al., 2021; Sunaga-Franze et al., 2021). The addition of enzymes necessitates an appropriate incubation temperature, such that the enzymes are in the most suitable state for digestion. However, cellular machinery is also active at the appropriate temperature for enzymatic action, leading to alterations in gene expression. A few scientists have found that when obtaining single cells from animals, the addition of enzymes at the incubation temperature can lead to a dramatic increase in gene expression after incubation at 37 °C (Adam et al., 2017; van den Brink et al., 2017; Potter, 2018; Denisenko et al., 2020). Similar results were obtained when plant cells were digested to obtain protoplasts. Isolating plant nuclei for single-cell transcriptomics would not face this challenge, as no enzymes are used and incubation is performed on ice, avoiding the activation of gene expression. Therefore, from the perspective of introducing artefacts and anomalous gene expression, plant nucleus-based single-cell transcriptomics sequencing is more precise, convenient, and applicable than protoplast-based single-cell transcriptomics. Researchers have also developed methods to overcome the limitations of protoplast release, such as performing independent bulk RNA sequencing experiments to eliminate the effect of enzyme addition or using transcription inhibitors (Potter, 2018; Denyer et al., 2019; Sunaga-Franze et al., 2021). Specifically, α-amanitin inhibits transcription via RNA polymerase II, thereby preserving gene expression patterns (Potter, 2018). Some researchers have extracted RNA from protoplast and unprotoplast plant tissues, identified genes induced by protoplast, and removed them from analyses (Denyer et al., 2019; Jean-Baptiste et al., 2019; Shulse et al., 2019). Protoplast-based single-cell transcriptome methods are not applicable, whereas nucleus-based techniques can. Protoplasts are used for plant single-cell transcriptomics; however, the methods for isolating protoplasts differ for different plant materials, even for the same plant and their exact position in the plant. As mentioned above, this is mainly because many factors affect the enzyme solution and incubation conditions adopted in the experiments. The main components of cell walls are similar, but differences in the functions of cells result in the production of distinct cell wall polymers surrounding each cell. Therefore, cell walls are considered heterogeneous when comparing plant cells of different types and developmental stages (Somssich et al., 2016). Thus, the methods for obtaining protoplasts lack applicability, leading to limitations in protoplast-based single-cell transcriptomics. In addition, these protoplast-based methods rely heavily on the quality and quantity of protoplasts and their compatibility with sequencing systems (Thibivilliers et al., 2020). Protoplast suspensions need to contain more intact protoplasts and less or no cell debris and damaged cells to avoid low cell numbers and quality, mRNA cell leakage, and obstruction of microfluidic systems, thereby ensuring that the microfluidic systems run correctly (Conde et al., 2021). Nucleus-based single-cell transcriptomics can overcome these problems owing to the introduction of a general method for plant nucleus isolation, which is an alternative to protoplast isolation methods and has been tested for its applicability to several different types of plants (Sunaga-Franze et al., 2021).

The applicability of these two procedures is not only reflected in isolation techniques, but also in the requirements for cell sequencing, such as the size of sequencing materials or freshness of sequencing samples (Hwang et al., 2018; Denisenko et al., 2020; Nadelmann et al., 2021). Generally, to avoid obstruction of sequencing systems, the sequencing platform restricts the cell size. The protoplast was larger than the nucleus, which increased the possibility of obstruction. The freshness of protoplasts is also critical for sequencing, and it is necessary to maintain their shape to avoid membrane breakage. However, nuclei can be obtained from any plant cell, regardless of its freshness and size, and even from frozen plant tissues (Krishnaswami et al., 2016; Lake et al., 2016; Bakken et al., 2018; Wu et al., 2019; Nadelmann et al., 2021). Protoplast-based sequencing is a stringent requirement for sequencing materials.

Nuclei seem to be more appropriate for single-cell transcriptomics than protoplasts; however, isolating nuclei from plant cells results in the loss of cell cytoplasm, chloroplasts, and mitochondria, implying that single-nucleus RNA-seq (snRNA-seq) usually contains less transcription information. Therefore, snRNA-seq has limitations in obtaining information from particular cell types because it only uses a part of the cell, that is, the nucleus, instead of an entire cell for transcriptional profiling (Hu et al., 2018; Maitra et al., 2021). The cell nucleus contains only 10–20% of all cellular transcripts; therefore, nuclear RNA does not entirely reflect the RNA from the whole cell, which may increase the difficulties in the analysis (Bakken et al., 2018; Potter, 2018; Nadelmann et al., 2021). Moreover, snRNA-seq excludes critical gene information from outside the nucleus and does not completely represent the single-cell transcriptome. From this point of view, protoplasts are more accurate than plant nuclei for single-cell transcriptome analysis. However, in research on frozen samples, precious frozen samples, complex plant tissues that are difficult to digest, and those that are easily influenced by gene expression changes during protoplast digestion, plant nuclei are still more appropriate for sequencing.

The most significant difference between single-cell and spatial transcriptomics is that the former is highly resolved but loses spatial information, whereas the latter preserves spatial information but fails to achieve precision. Therefore, if more detailed research is preferred, such as further exploration of cell types and their molecular characteristics in response to different environmental stimuli, single-cell transcriptomics is often used. If the characteristics of different spatial regions or the molecular characteristics of cells in a certain region of plant organs are preferred, for example, by constructing a spatiotemporal atlas of the development of floral organs, spatial transcriptomics is often used. Microfluidic technology is typically used to separate single cells in single-cell transcriptomics. Microarray technology is typically used to store spatial information in transcriptomics. Each spot on the microarray has millions of oligonucleotide probes. An oligonucleotide probe sequence used in spatial transcriptomics usually has a spatial barcode to store spatial information, whereas the barcode in single-cell transcriptomics is used to distinguish different cells (Gurazada et al., 2021).

## 7 Challenges and perspectives

Single-cell transcriptomics is a mature analytical technology in animal research, but a rising and naive front field in plant research, which still needs further exploration and development. Earlier sections revealed problems in both protoplast-based and plant nucleus-based single-cell transcriptomics, and these difficulties need to be urgently solved. In contrast, this is the era of multi omics, and finding ways to combine single-cell transcriptomics with other omics is noteworthy. Integrating single-cell transcriptome analysis with other single-cell technologies or omics technologies may provide a list of cell types, and more importantly, new insights into the regulatory logic and spatial organisation among cells (Stuart et al., 2019).

Plant materials contain different cell types, a few of which are resistant to digestion owing to their secondary cell walls, implying that, based on currently used methods, such cells pose challenges in protoplast isolation, causing a biased analysis of cell types (Bezrutczyk et al., 2021; Conde et al., 2021; Farmer et al., 2021). The different positions of cells in plant materials also introduce differences in their area exposed to digestive enzymes; for example, a few cells located inside the plant materials and surrounded by other cells are challenging to digest. These cells are readily missed during the analysis because they are removed during filtration. A few groups have discovered that central stele cells are more difficult to capture than epidermal cells; thus, the preparation of plant tissue protoplasts is biased for cells located on the outer surface of plant materials and sufficiently exposed to digestion enzymes (Denyer et al., 2019; Jean-Baptiste et al., 2019; Shulse et al., 2019; Farmer et al., 2021). In addition, a few groups have observed that not only the position of cells, but also their developmental stage influences digestion, as younger cells are more readily digested than mature cells, leading to increased representation in the analysis (Jean-Baptiste et al., 2019; Ryu et al., 2019). Therefore, a few general and effective methods are needed to isolate protoplasts that can avoid errors caused by cell types, relative positions of cells, or developmental stages.

In addition, single-cell sequencing technology requires improvement to achieve more precise sequencing results. For example, droplet-based methods such as the 10× Chromium method help generate an RNA-seq library. In this method, a single cell or nucleus is encapsulated inside a gel bead that can supply a barcoded oligonucleotide for reverse transcription. The droplet-based method can be used to analyse thousands of cells in a single experiment. However, in a few cases, there is more than one cell or nucleus inside a droplet or a suboptimal number of beads inside a droplet, thereby causing problems in the analysis. One group tested the probability of this event and observed that approximately 20% of the droplets contained either no beads or more than one bead in their experiments (Lareau et al., 2020; Sunaga-Franze et al., 2021). Nanowell-based systems, such as Takara iCELL8, can also generate an RNA-seq library and do not suffer from this problem because they capture single cells or nuclei in a nanowell. Researchers can check the number of cells or nuclei by using microscopy. However, the chips used in this method have only a fixed number of nanowells, resulting in limited scalability (Sunaga-Franze et al., 2021). Therefore, it is important to improve the relevant technology to ensure that one droplet contains one bead and one cell or nucleus and provide better scalability.

In single-cell transcriptomics, cells are digested and isolated from their original position in the tissue, and their spatial information is lost. Scientists have successfully mapped scRNA-seq data by inferring cellular sources and locations from scRNA-seq data and *in situ* RNA patterns (Achim et al., 2015; Satija et al., 2015). Scientists have also constructed a 3D atlas in plant research by combining scRNA-seq and microscopy-based 3D spatial reconstruction (Neumann et al., 2022). Recent studies have used spatial transcriptomics to preserve spatial information. Single-cell transcriptome analysis can be strengthened by combining it with a spatial transcriptome analysis. The cell types of whole plant tissues can be classified using single-cell transcriptome analysis, and the spatial information of whole tissues can be obtained using a spatial transcriptome. Researchers have established an *in situ* single-cell spatial transcriptome method in plants based on stereo-seq and applied it to study *A. thaliana* leaves (Chen et al., 2022; Xia et al., 2022). Their method differed from the single-cell transcriptome methods described, but was similar to spatial transcriptome methods. They sectioned frozen plant tissues and adhered these plant sections to a stereo-seq chip for downstream operations. There are a few other integration strategies, and Longo et al. identified two main types of algorithms: deconvolution and mapping (Longo et al., 2021). Methods based on deconvolution algorithms separate discrete cellular subpopulations from the mRNA transcript mix at each capture point based on single-cell data. SPOTlight is such a method, integrating single-cell and spatial transcriptome, centering on a seeded regression using non-negative matrix factorisation, initialising with cell type marker gene, and non-negative least squares, and deconvoluting capture locations (Elosua-Bayes et al., 2021). There are other algorithms for deconvoluting spatial information, such as dampened weighted least-squares, negative binomial distribution, and Poisson distribution models (Andersson et al., 2020; Dong and Yuan, 2021; Cable et al., 2022). Some scientists believe that single-cell transcriptome data and spatial transcriptome data follow a particular probability distribution, and based on these hypotheses, a few probabilistic models have been proposed to help integrate these two types of data. Mapping methods combine these two types of transcriptome data, mapping the designated scRNA-based cell subtypes to each cell on a high-plex RNA imaging map, and mapping each scRNA-seq cell to a specific niche or region of tissue. Similar to deconvolution methods, a few mapping methods use probabilistic models, such as the variation Bayesian mean-field approximation (Qian et al., 2020). In addition, other methods, such as CellTrek, combine single-cell and spatial transcriptome data using co-embedding and metric learning approaches (Wei et al., 2022).

Similar to single-cell transcriptomics, research on spatial transcriptomics is more prevalent in mammals than in plants, partially because of differences between mammalian and plant cells. Different plant cell structures and organelles, including cell walls, vacuoles, chloroplasts, and the presence of secondary metabolites, make it challenging to obtain spatial transcriptomic data from plant cells or lead to cryosectioning problems (Bourgaud et al., 2001; Cosgrove, 2005; Giacomello et al., 2017; Gurazada et al., 2021). Specifically, owing to the cell walls, plant cell sections can neither be extremely thick nor extremely thin to avoid reducing mRNA release or capture and causing sectioning artefacts or low transcript levels (Gurazada et al., 2021). In addition, because a few plant tissues, such as leaves and roots, have curvatures, many sections are required to obtain complete information. Currently, there are few solutions to the challenges caused by these differences, and it is often necessary to optimise the method for specific plant species or tissues (Giacomello et al., 2017; Giacomello and Lundeberg, 2018; Gurazada et al., 2021). Furthermore, challenges have been introduced by spatial transcriptome technology, including resolution, sensitivity, throughput, and accessibility (Rao et al., 2021).

In addition, the related applications of single-cell transcriptomics and spatial transcriptomics can be explored further, whether using one of these two techniques or in combination with other analytical techniques. For example, although there has been comprehensive research on the root cell types of plants such as *A. thaliana* and *O. sativa*, there is almost no relevant research on the cross-species analysis of roots at the single-cell level. This cross-species analysis may provide a basis for analysing differences in elemental absorption among different plants. Relevant animal studies have identified a new cell subtype, defined signature genes, and revealed differences in drug absorption among species through cross-species analysis (Li et al., 2022). Consideration should be given to the spatial distribution of elements in plants to capture thorough spatial information. Combining single-cell and spatial transcriptome analyses with the distribution patterns and speciation of one or more plant tissue elements offers several opportunities, particularly for studying the effects of stress caused by these elements. In recent years, techniques for analysing elemental distribution and speciation in plant materials have been developed, making it possible to draw an element-based map with high resolution (Lu et al., 2013; Lu et al., 2017). The combination of these techniques will provide new insights into plant research. For example, a combination of these three techniques could be used to explore the effects of different elemental stresses on plant roots. Analysis of the elemental distribution would reveal the characteristics of the absorption of this element and its storage and transportation mechanisms in plant roots. Spatial transcriptome and single-cell transcriptome analyses help analyse the effect of this element on gene expression in plant roots, or the difference in absorption and storage caused by the difference in gene expression, to provide a gene reference for cultivating low-accumulation plant varieties.

## 8 Conclusion

Both single-cell and spatial transcriptome analyses are helpful but still developing technologies in plant research, although they are relatively mature in mammalian systems. Single-cell transcriptomics provides insights into each cell type in plant tissues, such as classifying the cell type and analysing the cell developmental trajectory, and there is likely to be more research investigating functional genes in the future. Some problems in plant single-cell transcriptomics are common to single-cell transcriptomics in all organisms, such as those in sequencing techniques, whereas other difficulties are unique to plant research, including the lack of standard methods for isolating protoplasts. Owing to differences in cellular features and culture conditions, digestion solutions, and incubation conditions for releasing protoplasts differ among studies, leading to a lack of applicability. The snRNA-seq method, which has several advantages over scRNA-seq using protoplasts, can overcome this problem; however, it lacks information outside the nuclei. Spatial transcriptome technology provides spatial information on targeted tissues; however, it fails to reach single-cell resolution. Therefore, in the future, the single-cell transcriptome technique requires more effort to increase the applicability of methods for releasing protoplasts, and the spatial transcriptome technique requires more effort to achieve high resolution. More importantly, the application of single-cell transcriptomics and spatial transcriptomics needs to be explored, such as cross-species analysis using single-cell transcriptomics in the roots of different plants, and the combination of single-cell analysis and other analyses, such as spatial transcriptome analysis and spatial element distribution analysis, also needs to be emphasised to compensate for their limitations and create ample possibilities for research.

## Author contributions

CC and LL conceived the manuscript. CC wrote the manuscript. YG and LL reviewed and revised the manuscript. All the authors contributed to the manuscript and agreed to the submitted version.
